# COVID-19 Drug Discovery Using Intensive Approaches

**DOI:** 10.3390/ijms21082839

**Published:** 2020-04-18

**Authors:** Ayumu Asai, Masamitsu Konno, Miyuki Ozaki, Chihiro Otsuka, Andrea Vecchione, Takahiro Arai, Toru Kitagawa, Ken Ofusa, Masami Yabumoto, Takaaki Hirotsu, Masateru Taniguchi, Hidetoshi Eguchi, Yuichiro Doki, Hideshi Ishii

**Affiliations:** 1 Center of Medical Innovation and Translational Research, Osaka University Graduate School of Medicine, Suita, Yamadaoka 2-2, Osaka 565-0871, Japan; aasai@cfs.med.osaka-u.ac.jp (A.A.); mkonno@cfs.med.osaka-u.ac.jp (M.K.); moz@cfs.med.osaka-u.ac.jp (M.O.); cot@cfs.med.osaka-u.ac.jp (C.O.); t.arai@unitech-op.com (T.A.); toru@kyowakai.com (T.K.); oof21443@ideacon.co.jp (K.O.); yabumoto.masami@gmail.com (M.Y.); hirotsu@hbio.jp (T.H.); heguchi@gesurg.med.osaka-u.ac.jp (H.E.); ydoki@gesurg.med.osaka-u.ac.jp (Y.D.); 2 Institute of Scientific and Industrial Research, Osaka University, 8-1 Mihogaoka, Ibaraki, Osaka 567-0047, Japan; taniguti@sanken.osaka-u.ac.jp; 3 Department of Gastroenterological Surgery, Graduate School of Medicine, Osaka University, Suita 565-0871, Japan; 4 Artificial Intelligence Research Center, Institute of Scientific and Industrial Research, Osaka University, 8-1 Mihogaoka, Ibaraki, Osaka 567-0047, Japan; 5 Department of Clinical and Molecular Medicine, University of Rome “Sapienza”, Santo Andrea Hospital, via di Grottarossa, 1035-00189 Rome, Italy; andrea.vecchione@uniroma1.it; 6 Unitech Co., Ltd., Kashiwa 277-0005, Japan; 7 Kyowa-kai Medical Corporation, Osaka 540-0008, Japan; 8 Prophoenix Division, Food and Life-Science Laboratory, Idea Consultants, Inc., Osaka-City, Osaka 559-8519, Japan; 9 Kinshu-Kai Medical Corporation, Osaka 558-0041, Japan; 10 Hirotsu Bio Science Inc., Tokyo 107-0062, Japan

**Keywords:** COVID-19, coronavirus, infection, drug discovery, drug repositioning

## Abstract

Since the infectious disease caused by severe acute respiratory syndrome coronavirus 2 (SARS-CoV-2) was reported in China during December 2019, the coronavirus disease 2019 (COVID-19) has spread on a global scale, causing the World Health Organization (WHO) to issue a warning. While novel vaccines and drugs that target SARS-CoV-2 are under development, this review provides information on therapeutics which are under clinical trials or are proposed to antagonize SARS-CoV-2. Based on the information gained from the responses to other RNA coronaviruses, including the strains that cause severe acute respiratory syndrome (SARS)-coronaviruses and Middle East respiratory syndrome (MERS), drug repurposing might be a viable strategy. Since several antiviral therapies can inhibit viral replication cycles or relieve symptoms, mechanisms unique to RNA viruses will be important for the clinical development of antivirals against SARS-CoV-2. Given that several currently marketed drugs may be efficient therapeutic agents for severe COVID-19 cases, they may be beneficial for future viral pandemics and other infections caused by RNA viruses when standard treatments are unavailable.

## 1. Introduction

Since an unusual type of pneumonia, which was distinct from common pneumonia in symptoms and lethality, was reported in Wuhan, China, in December 2019, nations across the globe have paid attention to this new infectious disease. On 12 January 2020, the World Health Organization (WHO; https://www.who.int) temporarily designated the virus causing this disease as the 2019 novel coronavirus (2019-nCoV). On February 11, 2020, the WHO officially renamed this infectious disease coronavirus disease (COVID-19). The coronavirus study group within the International Committee on Taxonomy of Viruses also renamed 2019-nCoV, as severe acute respiratory syndrome coronavirus 2 (SARS-CoV-2). At present, the COVID-19 pandemic is spreading all over the world, with cases reported in China [1] and 168 other countries, areas, and territories. As of 20 March 2020, the COVID-19 disease caused 8778 deaths as noted by the WHO (https://www.who.int).

To fight against this pandemic, scientists and healthcare workers have started to share their knowledge. Given the rapid spread of COVID-19 and the smaller timeframe available for developing new therapies, drug repurposing may be an ideal strategy that allows healthcare workers to treat COVID-19 using previously approved or investigational drugs [2]. Here, we gathered information that may be pertinent to drug discovery for COVID-19 via a systemic review of the PubMed database (https://www.ncbi.nlm.nih.gov/pubmed) from 2000 to 2020.

We searched the papers with “corona”, “COVID”, “MERS” and “SARS” as keywords. The publications that were described as the concerning biological characteristics, interaction with human or *Homo sapiens*, therapeutic targets, and therapeutic medications for their viruses, are included in this review from 2000 to 2020.

Since some information is protected by patents, this article surveyed published and shared information to establish a therapeutic strategy against COVID-19.

## 2. Currently Undergoing Clinical Studies for COVID-19

Several drugs, such as chloroquine, favipiravir, remdesivir and umifenovir, are currently undergoing clinical trials to test their efficacy and safety in the treatment of COVID-19. Most of these studies are currently taking place in China [3,4].

### 2.1. Favipiravir (Avigan, T-705)

Favipiravir has been developed as an anti-influenza drug and is licensed as an anti-influenza drug in Japan [5]. One of the unique features of favipiravir is its broad-spectrum activity against RNA viruses, including influenza virus, rhinovirus and respiratory syncytial virus. Previous studies demonstrated that favipiravir is effective at treating infections with Ebola virus, Lassa virus and rabies, and against severe fever with thrombocytopenia syndrome [5]. However, favipiravir is not effective against DNA viruses.

**Figure 1:**
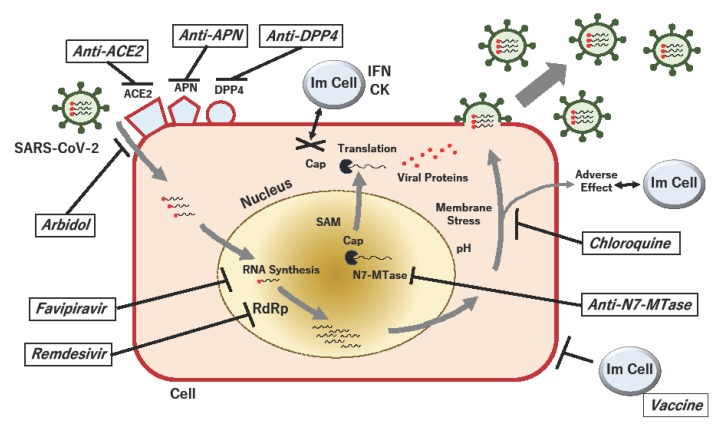
Proposed acting points of anti-SARS-CoV-2 in the replication cycle of the virus. When SARS-CoV-2 particles bind to their receptors, such as angiotensin-converting enzyme 2 (ACE2), aminopeptidase N (APN; CD13) and dipeptidyl peptidase 4 (DPP4; CD26), viral RNA is passed to the host cell, and RNA-dependent RNA polymerase (RdRp) produces viral RNAs. During RNA methylation, the RNA cap is formed, which protects against the host innate immune response, which involves the secretion of interferons (IFNs) and cytokines (CKs). The viral (guanine-N7)-methyltransferase (N7-MTase) plays a critical role in RNA capping, using the methyl donor S-adenosyl-methionine (SAM). The process of viral RNA synthesis and the translation of proteins is associated with pH-dependent membrane stress, which can elicit adverse effects against immune and non-immune cells. If the viral replication cycle is not inhibited and infected cells are not eradicated, packed viruses will be disseminated to other cells in the host. Proposed drugs and their possible acting points against COVID-19 are shown by bold lines.

With regard to its mechanism, it is reported that favipiravir antagonizes viral RNA synthesis by acting as a chain terminator at the site where the RNA is incorporated into the host cell. By contrast, oseltamivir (Tamiflu), a neuraminidase inhibitor, blocks the cleavage of sialic acid and the subsequent entry of the virus into the cell [5]. Importantly, favipiravir, unlike oseltamivir, does not seem to generate resistant viruses [5]. This property of favipiravir suggests a potential benefit in the treatment of critical infectious diseases such as COVID-19 (Figure 1).

### 2.2. Remdesivir (GS-5734)

Remdesivir is a nucleotide analog that is used for the treatment of infections caused by the Ebola virus and the Marburg virus [6]. However, it has also shown activity against respiratory syncytial virus, Junin virus, Lassa fever virus, Nipah virus, Hendra virus, and the MERS and SARS coronaviruses [7,8,9].

Remdesivir inhibits RNA-dependent RNA polymerases, most likely through the delay of RNA chain termination in the cell [10]. It is therefore one of the most promising compounds for treating COVID-19 [4].

### 2.3. Umifenovir and Lopinavir/Ritonavir

Umifenovir (ethyl-6-bromo-4-[(dimethylamino)methyl]-5-hydroxy-1-methyl-2-[(phenylthio)methyl]-indole-3-carboxylate hydrochloride monohydrate; trade name Arbidol) is a potent Russian-made broad-spectrum antiviral agent, that is used to treat influenza A and B viruses and hepatitis C virus (HCV) [11]. Although the mechanism slightly differs depending on the virus, it is reported that umifenovir inhibits viral fusion with the host cell membrane and subsequent entry into the host cell [11].

Recently, a trial involving the use of lopinavir/ritonavir (LPV/r), which are protease inhibitors used to treat HIV, in adults hospitalized with severe COVID-19, showed no observable benefit of LPV/r treatment beyond the standard of care [12].

Another retrospective cohort study tested umifenovir combined with LPV/r, versus LPV/r alone, against COVID-19 [13]. The results showed a favorable clinical response with umifenovir and LPV/r compared to LPV/r alone [13]; nevertheless, further studies will be necessary to determine efficacy and the occurrence of resistance. Since SARS-CoV-2 needs to undergo activation on the cell surface, umifenovir combined with LPV/r will help prevent the entry of the virus. The identification of more specific mechanisms may be beneficial for future clinical applications.

### 2.4. Chloroquine Phosphate

It was reported that chloroquine phosphate, a well-established drug used to treat malaria, was shown to have apparent efficacy, and was acceptably safe, when used against COVID-19 in multicenter clinical trials conducted in China [14]. In China, it was recommended that chloroquine phosphate be included in the next version of the Guidelines for the Prevention, Diagnosis, and Treatment of Pneumonia Caused by COVID-19 issued by the National Health Commission of the People’s Republic of China [14].

Chloroquine, which has been used since 1934, has several anti-inflammatory and antiviral effects that have been reported by previous studies [15]. For instance, chloroquine exerts direct antiviral effects by inhibiting the pH replication of several viruses, including flaviviruses, coronaviruses, and retroviruses such as HIV [15]. Moreover, it is reported that chloroquine has immunomodulatory effects that involve decreasing the production and release of tumor necrosis factor-α (TNFα) and interleukin (IL)-6 [15].

During a viral infection, the immune response is activated and the production and release of pro-inflammatory cytokines, TNFα, IL-1, IL-6 and interferon-gamma (IFNγ) is increased. Chloroquine, however, blocks these events [15]. Accordingly, chloroquine also prevents further deleterious mechanisms that may lead to acute respiratory syndrome, such as the alteration of tight junctions, the further release of pro-inflammatory cytokines, and increases in microvascular permeability [15].

Previous studies showed that the inhibitory effects involve the inhibition of autophagy [16]. Autophagy is a response mechanism to cellular membrane stress, induced by nutrient deprivation, hypoxia, and exposure to radiation and chemotherapeutic agents [17]. In animal experiments, chloroquine is highly effective in treating avian influenza A H5N1 virus infection by inhibiting autophagy [16]. Since chloroquine and its analog hydroxychloroquine are clinically relevant inhibitors of autophagy [17], the application of chloroquine may be reasonable and facilitated.

A recent study using cancer stem cells demonstrated that mefloquine hydrochloride, an antimalarial drug used to treat patients with resistance against chloroquine, efficiently eliminated colorectal cancer stem cells by disrupting endolysosomal proteins RAB5/7 [18]. Given that this lysosomal-dependent mechanism is a common platform for viral infection [19], other inhibitors of autophagy may be worth examining for the treatment of emerging infectious diseases, such as COVID-19. In the context of drug repurposing for COVID-19, it is also suggested that resistance against inhibitors of autophagy may be worth further examination.

## 3. Targeting Potential Entry Mechanism of COVID-19

### 3.1. Angiotensin-Converting Enzyme 2 (ACE2)

Previous studies indicated that the angiotensin-converting enzyme 2 (ACE2) is the functional receptor for SARS-CoV [20], determined via single-cell sequencing [21] and the structural analysis of proteins [22].

The latter study demonstrated that the receptor-binding domain (RBD) of the viral spike (S)-protein in SARS-CoV-2 shows a strong interaction with human ACE2 molecules, despite its sequence diversity [21]. The authors also suggested that SARS-CoV-2 poses a significant public health risk for human transmission via the S-protein–ACE2 binding pathway [21]. Interestingly, the study showed that ACE2 was preferentially expressed by a small population of type II alveolar cells, and that males have higher ACE2 expression than females [1,21]. The study also suggests that the binding of SARS-CoV-2 to ACE2 will increase the expression of ACE2 [1].

In many human and rodent studies, ACE2 expression is induced by treatment with ACE inhibitors (ACEIs) or angiotensin II receptor blockers (ARBs), which are commonly used as antihypertensive drugs [23]. The expression of sodium-dependent neutral amino acid transporter B(0)AT1 depends on the presence of ACE2 in the respiratory tract [24]. Given that COVID-19 includes symptoms such as fever (98%), cough (76%), dyspnea (55%) and fatigue/muscle pain (44%) [1], its symptoms may be relevant to the respiratory expression of ACE2.

A recent retrospective study indicated that COVID-19 patients with cardiovascular disease (CVD) have a higher risk of mortality [25]. Lower lymphocyte counts and higher body mass indices (BMI) are more often seen in patients with serious conditions [25]. A recent study showed that the use of ACEIs or ARBs for treating CVD does not affect the morbidity and mortality of COVID-19 [25].

In addition, it has been reported that the small intestine is the organ expressing ACE2 most highly [23]. Given that SARS-CoV-2 can be detected in the excrement of COVID-19 patients [26,27], these observed cases might involve cells in the small intestine infected with the SARS-CoV-2 binding receptor.

The crystal structures of S-protein binding to ACE2 has been revealed as an important interaction between host and SARS-CoV-2 [28,29]. In addition, it is known that ACE2 binds to Angiotensin II receptor type 1 (ATR1) and the sodium-dependent neutral amino acid transporter B^0^AT1, also known as SLC6A19, and that their bindings affect the binding between ACE2 and S-protein [30,31]. Moreover, Phosphatidylinositol 3-phosphate 5-kinase (PIKfyve), two-pore channel subtype 2 (TPC2) and cathepsin L are important for entry into cells [32]. Among them, it was reported that SARS-CoV S murine polyclonal antibodies, targeting conserved S epitopes, inhibited SARSCoV-2 entry [33]. Many therapeutic targets in the entry pathway via ACE2 have been reported, meaning ACE2 would therefore be a promising target for therapy of SARS-CoV-2.

### 3.2. Dipeptidyl Peptidase 4 (DPP4; CD26)

It was reported that dipeptidyl peptidase 4 (DPP4) is a functional receptor for the emerging human coronavirus via S-protein, as well as ACE2 [34]. The interaction between the virus and the host cell membrane allows for viral S-protein-directed cell–cell fusion, and the resultant spread of viral infections [35]. As another example relevant to drug repurposing and the ideal strategy for confronting COVID-19, the specific role of DPP4 on COVID-19 remains to be investigated. Further research is necessary to utilize DPP-4 as a therapeutic target for COVID-19.

### 3.3. Aminopeptidase N (APN; CD13)

It was previously reported that aminopeptidase N (APN) is involved in broad receptor engagement, which promotes the cross-species transmission of COVID-19 [36].

Interestingly, previous studies identified APN as a surface marker for cancer stem cells in the human liver [37]. Repurposing previous studies also allowed for the development of a poly(ethylene glycol)-poly(lysine) block copolymer-conjugate (Ubenimex) that targets APN specifically [38]. As drugs that can be repurposed, low doses of APN inhibitors, including Ubenimex or its derivatives, may be beneficial for inhibiting the spread of the virus.

## 4. Control of Virus-Specific RNA Modification in COVID-19

Although the modification of RNA by methylation is critical in biology, methylation is also important for the process of RNA capping in coronaviruses [39].

Like the coronaviruses that cause SARS and MERS, the mechanism of RNA capping may also be a draggable target in SARS-CoV-2. RNA capping plays a role in the transcription of viral RNA, as well as stability, replication, and evasion from the host’s immune response.

Many RNA viruses, including the coronaviruses, have evolved mechanisms for generating their cap structures with methylation at the N7 position of the capped guanine, and the ribose 2’-*O*-position of the first nucleotide. This mechanism plays a critical role in pre-mRNA splicing, mRNA export [40], RNA stability (via the blocking of degradation by 5’-3’ exoribonuclease) [41], translation initiation (by promoting host eukaryotic translation initiation factor 4E (eIF4E) binding) [42], and escaping the host’s innate immune system [43].

In general, 5’-end-capped mRNAs are produced through several steps [39]. Although there is no evidence to demonstrate the existence of an RNA guanylyltransferase (GTase) that is unique to coronaviruses, the coronaviral (guanine-N7)-methyltransferase (N7-MTase) plays a role in processing RNA to produce the cap-0 structure (m^7^GpppN) [42] in the proceeding reaction by 2’-*O*-MTase, to form the cap-1 (m^7^GpppNm) and cap-2 (m^7^GpppNmpNm) structures [44]. Both N7-MTase and 2’-*O*-MTase are catalyzed via the transfer of a methyl group from S-adenosyl-methionine (SAM) to the RNA substrate through the DxGxPxG/A SAM-binding motif. During the methylation process, S-adenosyl-homocysteine (SAH) is generated as a byproduct.

Given the proposed function of the viral RNA cap structure in preventing 5’-triphosphate terminal (5’-pppN) from activating the host innate immune response (cap-0 and cap-1) [45,46,47,48], antagonizing the interferon-mediated antiviral response (cap-1) [43,49,50] and enhancing viral RNA translation (cap-0) [51], it is proposed that coronaviral N7-MTase will be an attractive target for new antiviral drugs [39]. It is suggested that drug discovery is just as worthwhile, in testing anti-viral activity, as the repositioning of drugs in the case of COVID-19.

## 5. Conclusions

Although specific treatments, including vaccines, have not yet been developed for COVID-19, effective prevention methods are now recommended on a global scale. Accordingly, to overcome this pandemic, developing specific inhibitors for viral entry and replication, as well as drug repositioning, will be necessary. As above, several clinical trials and drug repositioning studies are currently ongoing. Eventually, new studies will allow us to better control this pandemic and identify new treatments. Computational calculation and artificial intelligence would help the rapid development of a therapeutic method. On the other hand, accurate crystal structure determination and much drug-response data are necessary for its success. The efficient sharing of information will be important for overcoming this pandemic in the era of globalization.
